# Multimodal Monitoring in Eclamptic Intracerebral Hemorrhage Complicated by Reversible Cerebral Vasoconstriction Syndrome (RCVS): A Case Report

**DOI:** 10.1155/crcc/7731993

**Published:** 2026-08-27

**Authors:** Giada Cucciolini, Marta Pillitteri, Lara Vegnuti, Francesco De Masi, Baldassare Ferro

**Affiliations:** ^1^ Department of Surgical, Medical, Molecular Pathology and Critical Care Medicine, University of Pisa, Pisa, Italy, unipi.it; ^2^ Departmental Structure of Neuroanesthesia and Intensive Care, Azienda Ospedaliero-Universitaria Pisana, Pisa, Italy, ao-pisa.toscana.it; ^3^ Anesthesia and Critical Care Department, Azienda Toscana Nord Ovest, Livorno, Italy

**Keywords:** eclampsia, HELLP, multimodal monitoring, RCVS, TCCD

## Abstract

**Background:**

Eclampsia is a rare but severe obstetric condition associated with maternal morbidity and mortality. Neurological complications are rare and often compounded by systemic involvement such as HELLP syndrome and coagulopathy. In this complex setting, multimodal neuromonitoring may support individualized management.

**Case:**

A 32‐year‐old woman at 37 weeks′ gestation presented with new‐onset tonic–clonic seizures, severe hypertension, and laboratory findings consistent with HELLP syndrome and disseminated intravascular coagulation. Following emergency cesarean section, brain CT revealed a right frontal intracranial hemorrhage (ICH) with mass effect. Given the high surgical risk related to coagulopathy, conservative management was pursued after placement of an invasive intracranial pressure (ICP) monitoring. In the neuro–ICU, a multimodal monitoring strategy combining ICP waveform analysis, continuous electroencephalography, cerebral near‐infrared spectroscopy, and transcranial color‐coded duplex ultrasound (TCCD) guided therapy. Medical treatment successfully controlled intracranial hypertension and restored cerebral compliance. Daily TCCD examinations revealed progressively increased middle cerebral artery flow velocities, leading to early suspicion and radiological confirmation of reversible cerebral vasoconstriction syndrome (RCVS). Treatment with verapamil and levosimendan resulted in rapid normalization of Doppler velocities without systemic hypotension.

**Outcome:**

The patient showed progressive neurological recovery and was discharged to rehabilitation. After 6 months, functional outcome was favorable (modified Rankin Scale = 2).

**Conclusions:**

This case underscores the value of bedside TCCD integrated with multimodal neuromonitoring in the management of a complicated eclampsia with ICH and RCVS. Assessment of cerebral hemodynamics and perfusion may support safe conservative management in high‐risk neuro‐intensive care patients.

**Summary**

• The correct approach to manage complex cases of intracranial hypertension and systemic disease should be individualized, weighing the risk/benefit of medical and surgical treatments
• Multimodal neuromonitoring should be part of the toolbox of the neurointensivist
• Alterations of cerebral flow and autoregulation can be monitored by TCCD. The peculiarity of being noninvasive and easy to use makes it attractive to be used not only in neurointensive care unit but as a guide of treatment for all critical care patients.

## 1. Introduction

Despite its rare incidence, eclampsia is responsible for 15% of maternal death worldwide and for a three‐fold to 25‐fold increased risk of developing severe maternal and fetal complications [1].

The neurologic manifestations of eclampsia—namely, the new onset of clinical seizures in a patient with or without signs of pre‐eclampsia—represent only the tip of the iceberg of a complex systemic disease whose pathophysiology remains only partially understood.

The spectrum of neurological complications associated with eclampsia is broad, ranging from cerebral edema, posterior reversible encephalopathy syndrome (PRES), reversible cerebral vasoconstriction syndrome (RCVS), and ischemic stroke to intracranial hemorrhage (ICH), including intraparenchymal, subdural, basal ganglia, and pontine hemorrhages, as well as subarachnoid hemorrhage secondary to cerebral venous thrombosis.

These severe neurological conditions are further complicated by potentially life‐threatening systemic involvement, including HELLP syndrome (characterized by elevated liver enzymes and low platelet count), as well as varying degrees and forms of coagulopathy, renal dysfunction, and respiratory failure [2].

The neuro‐intensive care management of these conditions is complex and should be individualized, weighing the risks and benefits of eventual neurosurgical treatments and its associated complications in coagulopathy conditions.

At the same time, it is important to fully embrace the concept of the evolution and management of intracranial compartment syndrome. The potential of multimodal neuromonitoring—including neuroimaging, intracranial pressure (ICP) monitoring, transcranial color‐coded duplex ultrasound (TCCD), cerebral near‐infrared spectroscopy (cNIRS), continuous electroencephalography (cEEG), and pupillometry—allows clinicians to guide not only timely and aggressive pharmacologic treatment but also the overall clinical decision‐making process [3].

We report here the rare case of a young female presenting with eclampsia and severe rare neurological complications, such as ICH, intracranial hypertension, and RCVS, complicated by HELLP, kidney, liver, and respiratory failure. In this case, we describe how nonsurgical conservative management guided by a multimodal monitoring approach was a successful strategy to achieve an almost complete neurological recovery.

## 2. Case Presentation

**Figure 1:**
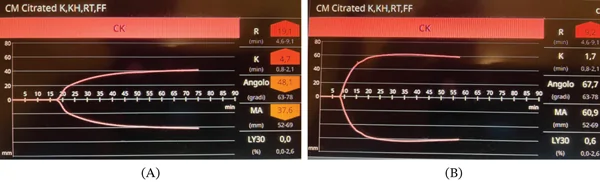
(A) Thromboelastography alterations at admission and (B) amelioration after blood products infusion.

A 32‐year‐old female patient presented to the emergency department at 37 weeks of gestational age for acute onset of tonic clonic seizures. At admission, her arterial blood pressure (ABP) was 190/110 mmHg, heart rate was 110 bpm, and SpO_2_ was 92% via a nonrebreather mask. Her anamnesis was negligible for previous relevant pathologies. No signs of pre‐eclampsia were referred during pregnancy, except for a mild increase of ABP in the last week (150/90 mmHg). Routine exams revealed a low platelet count (48 × 10^3^/*μ*L), D‐dimer (6000 pg/mL), fibrinogen (1.8 g/L), creatinine (1.64 mg/dL), and transaminases ALT (670 mU/mL), and AST (800 mU/mL). A diagnosis of eclampsia complicated by HELLP syndrome and disseminated intravascular coagulation (DIC) with hypocoagulable state was made. Hypocoagulable state was then confirmed by thromboelastography and corrected with infusion of platelets, fresh frozen plasma, and fibrinogen until normalization of the viscoelastic test (Figure 1).

High‐dose magnesium sulphate following Pritchard′s protocol was administered from admission until Day 2. Labetalol was also administered for heart rate and ABP control during these acute phases.

**Figure 2:**
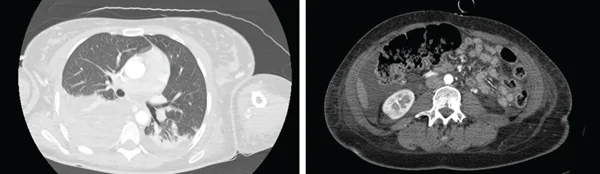
Thoracic and abdominal CT scan showing right pleural and peritoneal effusion.

**Figure 3:**
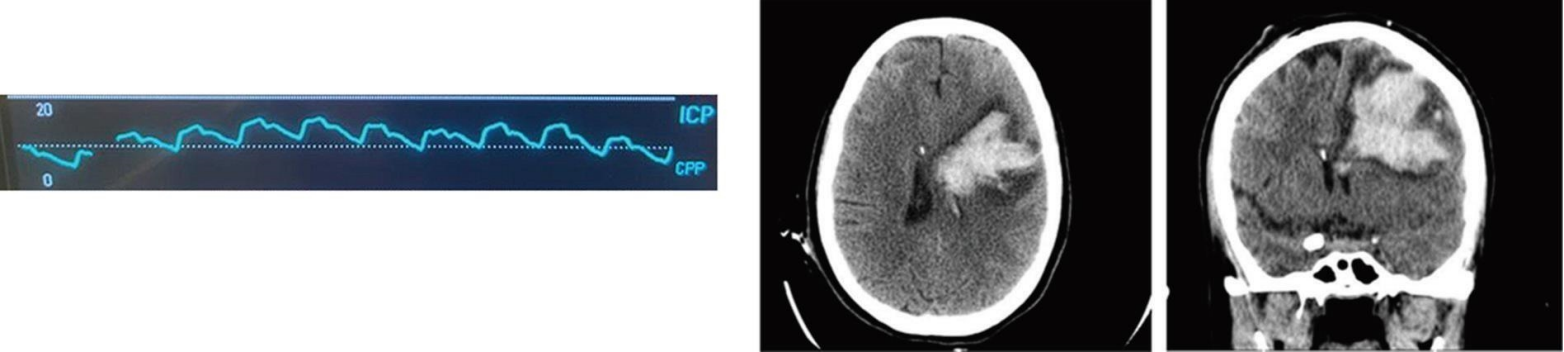
Normal ICP waveform and first head CT scan. Midline shift was 8 mm.

After the cesarean section, a total body CT scan was performed, revealing massive right pleural effusion and abundant peritoneal effusion with no signs of active bleeding or parenchymal lesions (Figure 2). The head CT scan showed a right frontal intraparenchymal hemorrhage (ICH) with perilesional edema causing mass effect with a midline shift of 8 mm, left ventricle compression, and subarachnoid hemorrhage with no hydrocephalus (Figure 3). CT angiography was negative for the presence of cerebral aneurysms, arteriovenous malformations, or cerebral venous sinus thrombosis.

An intraparenchymal ICP probe was then positioned. The first ICP registered value was 11 mmHg with a nonpathological waveform morphology (p3 < p2 < p1) (Figure 3) [4]. After a multidisciplinary evaluation, a watchful waiting approach was decided in accordance with the neurosurgeon on call due to the coagulation abnormalities secondary to the HELLP syndrome and a DIC state.

**Figure 4:**
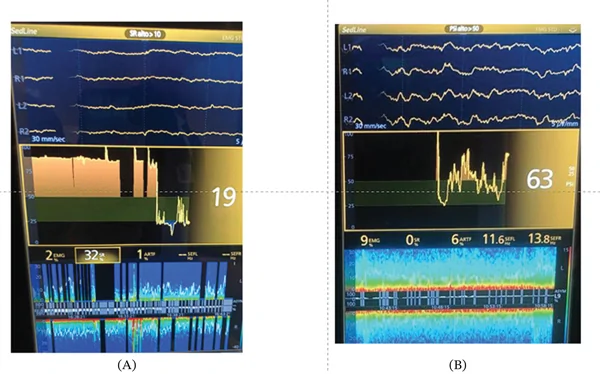
(A) Continuous frontal EEG monitoring (Sedline, Masimo, Irvine, United States) and spectral analysis to maintain a state of burst suppression. (B) Normal pattern without significant EEG alterations during awakening.

A multimodal monitoring strategy was started in the intensive care unit, combining the use of ICP, cNIRS (O_3_, Masimo, Irvine, United States), brain ultrasound (TCCD), and cEEG with density spectral array (Sedline, Masimo, Irvine, United States) (Figure 4A and Table 1). For the purpose of neuroprotection, the patient was kept deeply sedated under mechanical ventilation (burst suppression state monitored via EEG). An ultrasound‐guided thoracentesis evacuated 1 L of serohematic pleural effusion; protective mechanical ventilation was applied with a normocapnia target (pCO_2_ 35 mmHg, driving pressure 8 cmH_2_0, tidal volume 7 mL/kg, and mechanical power < 8 joules/min). Mean arterial pressure (MAP) was stable with a cerebral perfusion pressure > 80 mmHg without the need for hemodynamic support.

**Table 1:** Essential monitoring tools in multimodal neuromonitoring and their usefulness.

Monitoring tool	Meaning/usefulness
Invasive intracranial pressure probe	ICP monitoring is useful to assess not only the absolute value (that is of course important) but even other derived parameters. The relationship between the three components of the ICP waves (p1–p2–p3) changes with modifications in the intracranial compliance. In this case, the p1/p2 ratio was used to infer changes in the intracranial compliance. A full description of all the derivable parameters from the ICP is available in a recent review [5].
Pupillometry	Automated infrared pupillometry (AIP) provides a standardized, quantitative, and highly reproducible measurement of the pupillary light reflex (PLR), overcoming the significant interobserver variability inherent in manual examination. It measures pupil size, percentage of constriction, constriction velocity, dilation velocity, and latency and integrates them into the Neurological Pupil Index (NPi), a composite score ranging from 0 to 5, with values < 3 considered abnormal. A comparison study by Robba et al. found a significant but moderate inverse correlation between NPi and invasive ICP (*r* = −0.41), with an AUC of 0.71 for detecting intracranial hypertension (ICP > 20 mmHg) [6].
Brain sonography and optic nerve sheath diameter (ONSD)	Brain US is a bedside, repeatable, and noninvasive tool that allows assessment of cerebral physiology. It complements other neuromonitoring tools giving real‐time information on cerebral blood flow (i.e., brain perfusion), intracranial dynamics, and structural changes (e.g., hydrocephalus, midline shift, or new hemorrhages/masses). In addition, optic nerve sheath diameter (ONSD) ultrasonography can provide indirect information on intracranial pressure [7, 8].
	In our case, particularly, TCD was used to diagnose the vessel narrowing and vasospasm, as well as in suspect of an increased ICP (increase of the pulsatility index) [9–11]. Another application was the assessment of the autoregulation functioning, which in our case was impaired. Brain US can in fact provide a qualitative and snapshot autoregulation estimation [12].
	For what concerns ONSD, it has been demonstrated that in case of increased ICP the ONSD increases. The best cutoff value to discriminate for increased ICP has been proposed to be between 5 and 6 mm [8, 13].
Near‐infrared spectroscopy	Near‐infrared spectroscopy is a noninvasive, portable, continuous bedside monitoring technique that uses near‐infrared light (700–1000 nm) to measure regional cerebral oxygen saturation (rSO₂), reflecting the balance between cerebral oxygen supply and demand.
	Reduction in the rSO_2_ value occurs when the brain is hypoperfused; in our case, a reduction in the mean arterial pressure (and thus in the cerebral perfusion pressure) provoked a reduction in the cerebral rSO_2_, signaling brain hypoperfusion.
Raw and processed EEG	Electroencephalography (EEG) is a cornerstone of multimodal neuromonitoring in neurocritical care, providing real‐time assessment of cerebral electrical activity. Full‐montage EEG allows identification of nonconvulsive seizures and status epilepticus, assessment of background activity, detection of ischemia‐related changes, and prognostication following acute brain injury.
	Simplified EEG systems use a limited number of frontal electrodes and were originally developed to assess depth of anesthesia. In the neurocritical care setting, these devices offer continuous bedside monitoring when full EEG is unavailable or difficult to implement. Processed EEG indices (e.g., density spectral array) and raw frontal EEG waveforms provide information on sedation depth, burst suppression during therapeutic coma, and major changes in cerebral activity. Reduced‐channel EEG systems should be viewed as screening and trend‐monitoring tools rather than substitutes for conventional continuous EEG [3, 14].
Conventional radiology (in this case, CT scan)	Computed tomography (CT) remains the cornerstone of acute neurological assessment in critically ill patients, allowing rapid identification of intracranial hemorrhage, cerebral edema, hydrocephalus, mass effect, midline shift, and evolving ischemic injury. Serial CT examinations are frequently used to monitor disease progression and evaluate the response to therapeutic interventions. Advanced imaging techniques, including CT angiography and CT perfusion, further contribute to the assessment of cerebral vascular status and tissue perfusion. In our case, a plain CT scan was used to diagnose the presence of the intracerebral hemorrhage, and the CT angiography revealed the vessels narrowing, supporting the diagnosis of the cerebral reversible vasoconstriction syndrome.
Other tools in neuromonitoring, not available in the described case, might be cerebral microdialysis and tissue invasive partial pressure of oxygen. Several educational reviews have been published on the topic and are not in the scope of this case report [3, 15, 16].	

**Figure 5:**
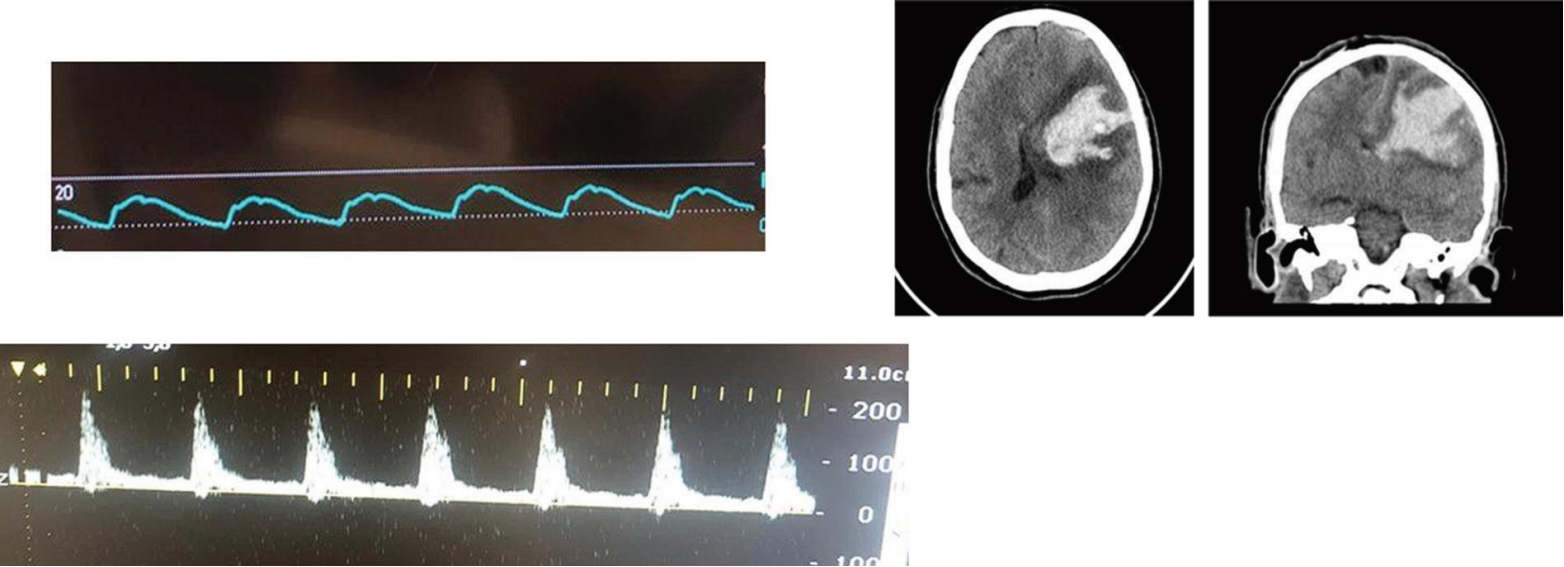
ICP waveform (blue line) with inversion of the p2/p1 ratio, decrease of diastolic flow, and pulsatility index > 1.4 (TCCD) at 24 h. A new CT scan was performed that revealed perilesional edema.

**Figure 6:**
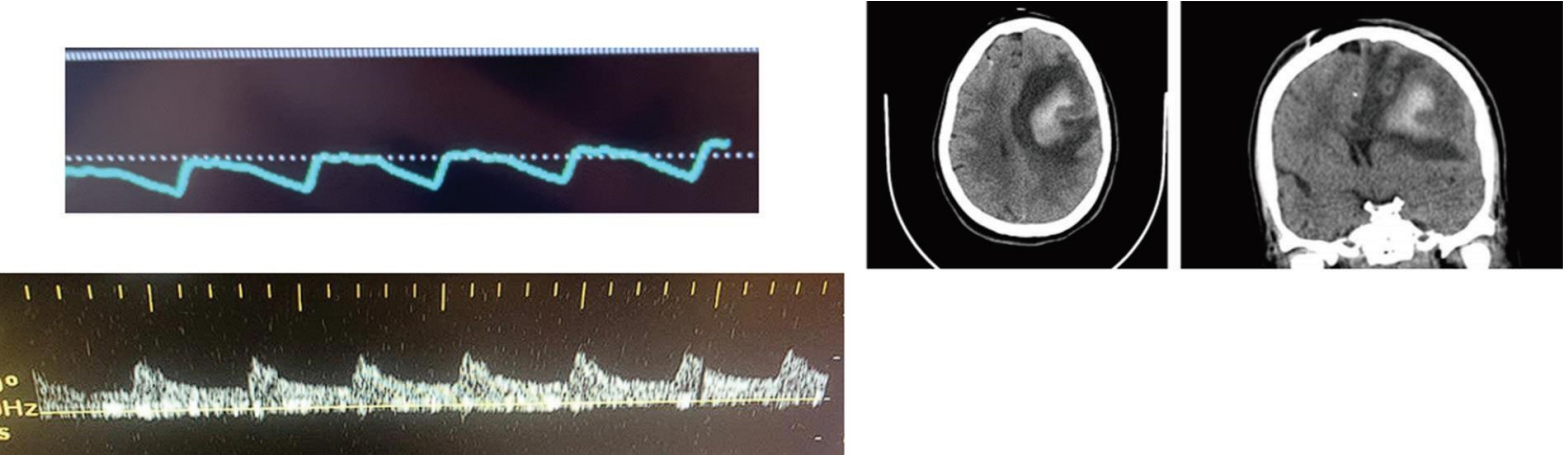
Normalization of the ICP waveform with p1 > p2 > p3. Recovery of the middle cerebral artery diastolic flow after aggressive medical therapy (TCCD, inferior panel). CT scan demonstrated natural evolution of the hemorrhage with reduction of the hematoma size.

**Figure 7:**
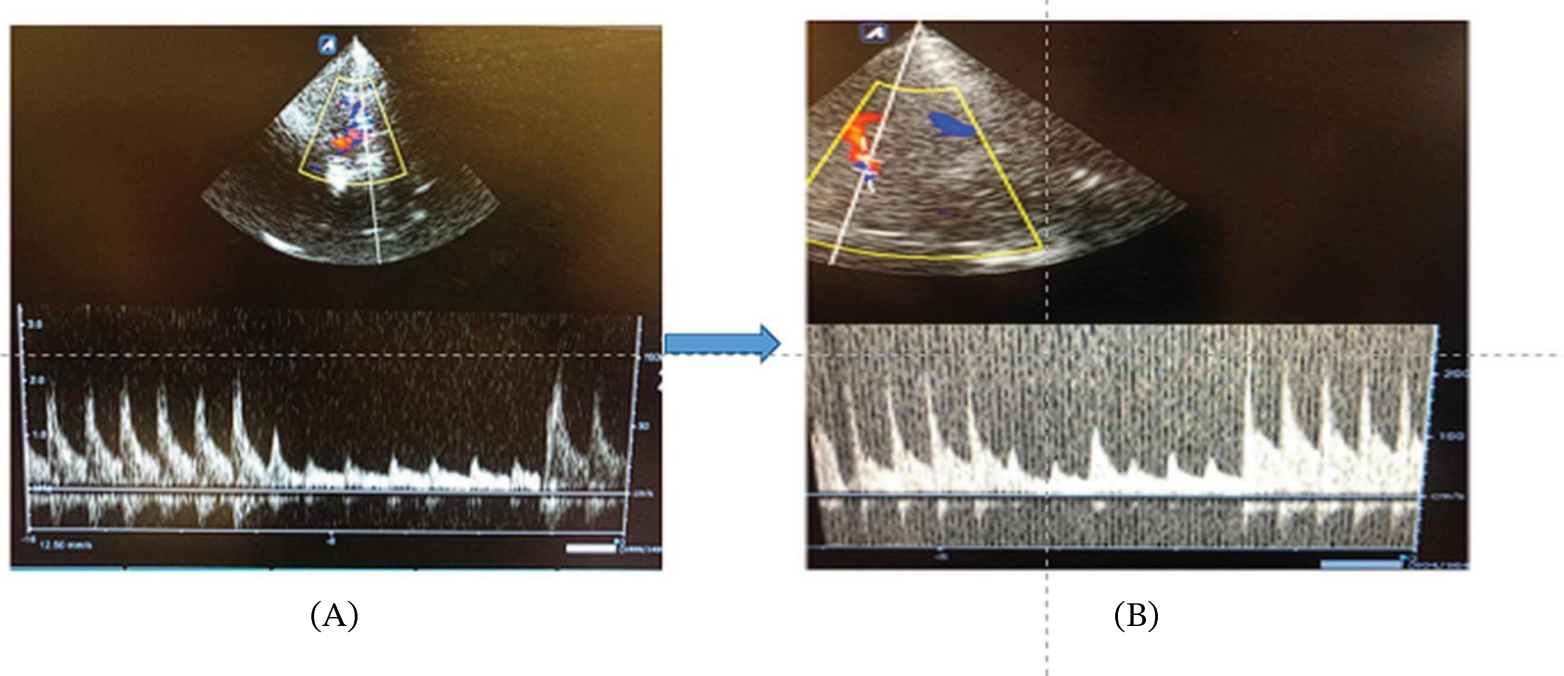
Evaluation of autoregulation with a transient hyperemic response test (THRR) with TCCD. After a brief occlusion of the carotid artery, flow recovery in the middle cerebral artery is analyzed. (A) At the beginning of the disease, the test is abnormal (flow velocity increase < 10% after occlusion). (B) After some days of therapy: restoration of cerebral autoregulation.

**Figure 8:**
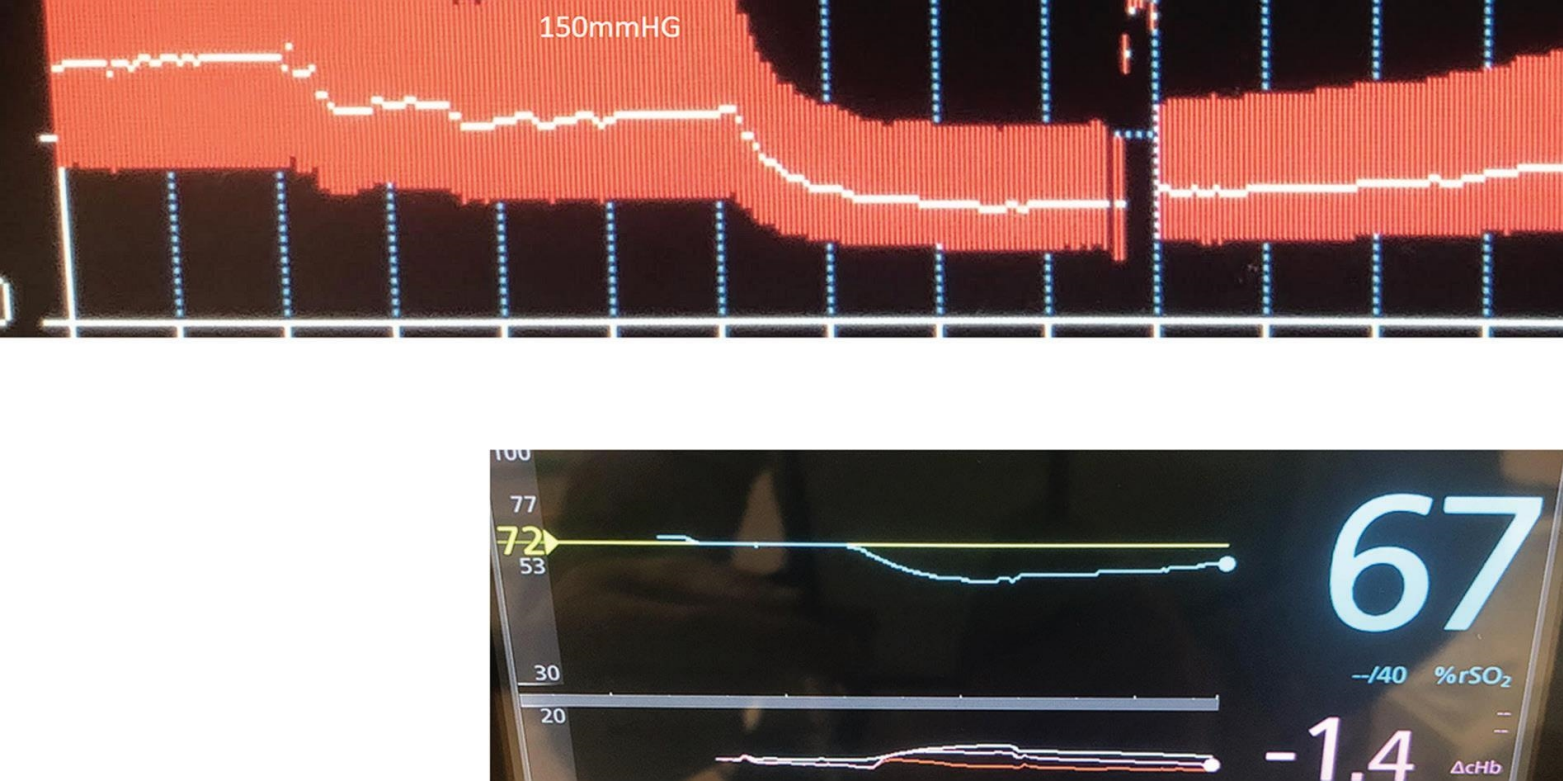
Correlation between variation of hemodynamic parameters and NIRS to maintain targets of cNIRS. Desaturation for estimated reduction of arterial systolic blood pressure of 150 mmHg and mean arterial pressure of 100 mmHg.

After 24 h, we observed an ICP increase (maximum value 25 mmHg) associated with waveform modification toward a less compliant intracranial system (p2 > p1). A new CT scan showed an increase of the cerebral edema without signs of rebleeding (Figure 5). To maintain a low ICP and a good cerebral perfusion pressure, aggressive anti‐edema therapy (boluses of mannitol 0.5–2 g/kg or hypertonic saline 1–4 mL/kg to target a cerebral perfusion pressure ≥ 65 mmHg and ICP ≤ 22 mmHg), strict control of temperature, body position (bed elevation 30°), and mild hypocapnia (30 mmHg) were used [17, 18]. TCCD revealed an increase in the pulsatility index (> 1.4) that normalized after administration of the medical therapy (Figure 6). Malfunctioning of cerebral autoregulation was diagnosed using the transient hyperemic response test (THRR) after carotid compression (Figure 7) [19]. Continuous NIRS showed a reduction of cerebral oxygenation when MAP fell below 100 mmHg (Figure 8).

After 96 h, ICP appeared more stable with a normalization of the p2–p1 ratio; a new cerebral autoregulation evaluation with THRR showed restoration of the physiological mechanism (Figures 3, 5, 6, 7B).

Anyway, daily‐based monitoring with TCCD was performed. Flow velocity in the right M1 cerebral artery progressively increased, reaching a maximum of 200 cm/s on the seventh day. A head CT angiography scan showed a multifocal narrowing in the cerebral vasculature with a beaded appearance localized especially in the right M1 segment and basilar artery. A diagnosis of RCVS was then made based on the clinical and radiological criteria outlined in Table 2 [20–23]. The RCVS2 score was computed in support for the diagnosis, yielding a score of 7 (scores ≥ 5 have a 90% sensitivity and 99% specificity for diagnosis) [24]. Combined use of oral verapamil (80 mg/daily) and slow infusion of levosimendan (2.5 mg in 24 h) was started to treat the vasospasm [25].

**Table 2:** Criterions for the diagnosis of CRVS. On the left, there are the clinical criterions; on the right, the application in our case.

Clinical and radiologic criterions for the diagnosis of cerebral reversible vasoconstriction syndrome	
1. Acute and severe headache (often thunderclap) with or without focal deficits or seizures	The symptom of headache was not identifiable as the patient developed the spasm while in coma for a cerebral hemorrhage.
2. Common triggers and risk factors: vasoactive substances (cocaine, amphetamines, SSRIs, ergot derivatives, triptans, cannabis, and nasal decongestants), postpartum state, physical exertion, sexual activity, migraine history, and female sex.	Our patient had a classic trigger that was the postpartum period.
3. Uniphasic course without new symptoms more than 1 month after clinical onset	No new symptoms were demonstrated by the patient during the rehab period nor in the following year.
4. Segmental vasoconstriction of cerebral arteries demonstrated by indirect (MRA or CTA) or direct catheter angiography	The CT angiography demonstrated a multifocal narrowing in the cerebral vasculature with a beaded appearance in the Willis polygon arteries, especially the right middle cerebral artery and the basilar artery.
5. No evidence of aneurysmal subarachnoid hemorrhage	No aneurysms were identifiable at the CT angiography.
6. Normal or near‐normal CSF (protein < 100 mg/dL, < 15 white blood cells per microliter)	The exam was considered not reliable due to the presence of blood into the CSF. A lumbar puncture was not performed. The infective source was excluded due to low white cell blood count, low C reactive protein, and absence of fever.
7. Complete or substantial normalization of arteries on follow‐up angiography within 12 weeks of clinical onset	A follow‐up CT angiography scan was executed 22 days after the first CT angiography. The exam demonstrated a regular caliper of the Willis polygon arteries. No vascular abnormalities were detected. A bilateral slight reduction of the intracavernous tract of the carotid syphon was the only abnormality detected.

**Figure 9:**
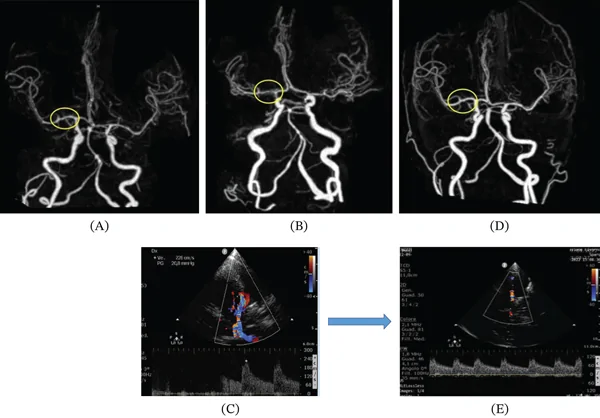
(A–B) CT angiography showing the vessel narrowing. (C) Increase of mean flow velocity in the right middle cerebral artery up to 200 cm/s at TCCD. (D–E) Normalization of flow velocities and vessels diameters after combined oral verapamil and levosimendan.

**Figure 10:**
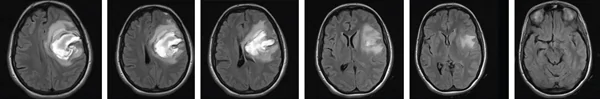
Head MRI scan showing stability of cerebral hemorrhage, edema, and midline shift.

NIRS and ICP levels were stable, and Doppler velocities normalized within 5 days (Figure 9D–E). MRI scan did not show any ischemic event and confirmed the stability of the known hemorrhagic lesion and cerebral edema (Figure 10).

## 3. Evolution, Outcome, and Follow‐Up

**Figure 11:**
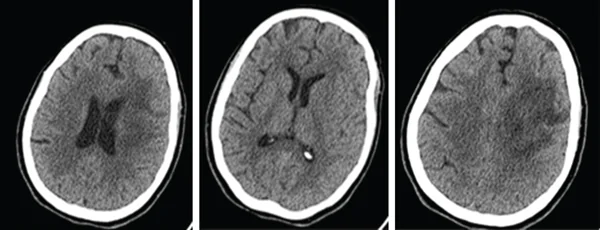
Head CT scan before discharge with normal evolution of the cerebral hemorrhage, resolution of midline shift, and re‐expansion of the left ventricle.

A percutaneous tracheostomy was performed on Day 11. After a gradual weaning from sedation, the patient regained consciousness without significant alterations in the EEG density spectral array (9B). The patient began a slow motor recovery, and after a rapid weaning from the ventilator, she was discharged to the rehabilitation center. At discharge, she was able to feed orally and move the upper and lower limbs spontaneously bilaterally. Last CT scan before discharge showed a normal evolution of the cerebral hemorrhage, resolution of the midline shift, and ventricular re‐expansion (Figure 11). The Willis polygon arteries had a regular caliper. No vascular abnormalities were detected. A bilateral slight reduction of the intracavernous tract of the carotid syphon was the only abnormality detected.

After 6 months, the patient had a modified Rankin Scale of 2 and a Cerebral Performance Category (CPC) score of 2. She had difficulties in reading and ideation and fine movements of the right hand.

## 4. Discussion

This rare case of eclampsia complicated by HELLP syndrome, ICH, and subsequent RCVS highlights the need for nuanced strategies when managing neurological complications in such critical situations. Although decompressive craniotomy or hematoma evacuation is reported in the literature to mitigate secondary brain injury from intracranial hypertension in eclampsia [26], surgical intervention carries significant risks in this cohort. Specifically, coagulation abnormalities compounded by arterial hypertension and secondary multi‐organ failure (hepatic and renal) present substantial risk factors for early rebleeding and hematoma expansion, even when utilizing thromboelastography‐guided transfusion algorithms [27]. While minimally invasive neurosurgical hematoma aspiration has gained recent interest for reducing mass effect and controlling ICP, this procedure has not yet been described in the literature for patients with eclampsia [28].

Recently, the novel paradigm of “intracranial compartment syndrome” has evolved beyond the reliance on rigid ICP thresholds to guide the management of intracranial hypertension [29]. This represents a shift away from the traditional, purely volumetric “mass‐effect” model dictated by the Monro–Kellie doctrine. Instead, it emphasizes that secondary brain injuries frequently stem from the disruption of endogenous neuroprotective mechanisms, such as cerebral autoregulation. This paradigm shift underscores the utility of multimodal neuromonitoring in detecting occult pathological alterations, including impaired intracranial compliance via ICP waveform analysis or cerebral desaturation via near‐infrared spectroscopy (NIRS) [3, 15]. Consequently, the intensity of medical management and the indications for surgical intervention are no longer dictated by predetermined therapeutic tiers or a superficial evaluation of absolute ICP values. Rather, treatment is tailored to restore cerebral compliance, blood flow, and perfusion, even in the absence of overt intracranial hypertension. Naturally, prompt surgical intervention remains indicated should medical optimization fail.

The combined use of TCCD and cNIRS has been explored for the diagnosis of an altered state of cerebral autoregulation; despite an imperfect correlation with the commonly used pressure reactivity index, TCD‐ and NIRS‐derived autoregulation indexes appear interesting to correlate and guide systemic and cerebral hemodynamics to maintain acceptable flow and oxygenation targets [30, 31].

In this case, daily assessment of flow velocities with TCCD has been of extreme value to make diagnosis, to monitor, and to follow the treatment of RCVS that is a prelude to insidious ischemic complications. RCVS is a medical condition in which there is multifocal arterial constriction and dilation in the cerebral vasculature and which may be associated with nonaneurysmal subarachnoid hemorrhage and ischemia [21]. Crucially, RCVS overlaps clinically with eclampsia, severe hypertension, and HELLP syndrome, as all present with acute severe headache, seizures, and cerebrovascular dysregulation [32]. In high‐acuity neuro‐obstetric presentations, severe hypertension, coagulopathy, and initial intracerebral hemorrhage can understandably dominate the diagnostic framework—exposing clinicians to the risk of diagnostic overshadowing. In such cases, persistent, atypical, or thunderclap headaches and evolving neurological findings are easily misattributed solely to eclampsia or the primary hemorrhagic event, leading to significant diagnostic delays. This case underscores that persistent or changing clinical features in the postpartum period must prompt active, ongoing reassessment. In this context, serial bedside TCCD combined with multimodal neuromonitoring serves as a crucial objective tool to detect evolving cerebral vasoconstriction early and prevent delayed recognition of RCVS. This condition correlates with pregnancy and exposure to certain drugs and can last even months with variable response to vasodilatory drugs. Angiographic intra‐arterial infusion of verapamil or nimodipine, or balloon angioplasty, has been rarely reported in the literature in awake patients with thunderclap headache secondary to RCVS and unresponsive to medical therapy [20, 25]. We decided to use an empirical off‐label combination of oral verapamil and intravenous levosimendan to avoid the hypotensive effect of the commonly used systemic nimodipine for vasospasm and its uncertain effect on ICP in a patient with a condition of alteration of cerebral autoregulation. Verapamil dosage was maintained under the reported references in literature (observational studies report commonly 120 mg/daily dosage [33]) to avoid hypotension and the need for higher doses of vasopressors. The rationale for levosimendan administration was based on its known vasodilatory properties and on a few supporting evidence suggesting a potential role in the treatment of cerebral vasospasm (although not in RCVS). Levosimendan induces vascular smooth muscle relaxation primarily through the opening of ATP‐sensitive potassium (KATP) channels leading to membrane hyperpolarization, closure of voltage‐gated calcium channels, reduction of intracellular calcium availability, and subsequent vasodilation [34]. Experimental and clinical studies have demonstrated that KATP channel activation promotes dilation of cerebral arteries and increases cerebral blood flow [35, 36], supporting a biologically plausible mechanism for the reversal of cerebral vasoconstriction. Nimodipine use was avoided in consideration of the hypotensive effect and its uncertain effect on ICP in a patient with a condition of alteration of cerebral autoregulation. This drug combination was able to rapidly decrease flow velocity and normalize vessels′ diameters in a short period. Levosimendan has been recently studied as a potential treatment of vasospasm in subarachnoid hemorrhage, and its preconditioning properties are of interest [25, 37]. This drug combination was able to rapidly decrease flow velocity and normalize vessels′ diameters in a short period of time.

Lastly, cEEG and its spectral analysis seem to become an essential monitoring system not only to treat and to prevent epileptic events but also to match oxygen delivery and consumption with sedation adjustments.

## 5. Limitations

This report has several limitations. First, as a single‐case experience from a single center, it cannot establish causal relationships or support the generalization of the proposed management strategy. Rather, the observations should be considered hypothesis generating.

Second, this patient presented with an exceptionally complex overlap of neurological and obstetric conditions, including eclampsia, HELLP syndrome, DIC, intracerebral hemorrhage, and RCVS. Management was therefore necessarily individualized and guided by the dynamic interaction of these coexisting conditions. Consequently, the diagnostic and therapeutic approach described here should not be directly extrapolated to patients presenting with any of these conditions in isolation.

Third, although normalization of cerebral blood flow velocities followed the initiation of oral verapamil and low‐dose levosimendan, the respective contribution of these treatments cannot be determined. RCVS is characterized by spontaneous reversibility, and the concomitant use of multiple therapeutic interventions precludes attribution of the observed improvement to either drug. In particular, the use of levosimendan represented an empirical, off‐label therapeutic choice based on pathophysiological considerations rather than established clinical evidence, and no conclusions regarding its efficacy can be drawn from this report.

Finally, the patient′s perspective was not available for inclusion in this report.

## 6. Conclusions

In conclusion, despite the inherent limitations of individual invasive or noninvasive monitoring modalities, this case underscores the vital role of integrating multimodal neuromonitoring and carefully interpreting its data to optimize the management of neuro‐intensive care patients. Randomized controlled trials aiming at demonstrating its potential benefit on the outcome and the development of new drugs for the treatment of vasospasm are urgently awaited.

## Author Contributions

Conceptualization: B.F. Writing – first draft: B.F., G.C., and M.P. Writing – review and editing: G.C., B.F., and F.D.M. Data curation: M.P., B.F., and L.V. Methodology: G.C. and B.F. Supervision: B.F.

## Funding

No funding was received for this manuscript. Open access publishing facilitated by Universita degli Studi di Pisa, as part of the Wiley ‐ CRUI‐CARE agreement.

## Disclosure

Validation: All the authors reviewed and approved the final version of the manuscript.

## Ethics Statement

Written informed consent was obtained from the patient for publication of this case report and its accompanying images. This study has been conducted in accordance with the Declaration of Helsinki (https://www.wma.net/wp-content/uploads/2016/11/DoH-Oct2008.pdf).

## Conflicts of Interest

The authors declare no conflicts of interest.

## Supporting information


**Supporting Information** Additional supporting information can be found online in the Supporting Information section. To improve reporting, the file has been provided in conjunction with the CARE (CAse REport) guidelines checklist [38].

## Data Availability

Data sharing not applicable to this article as no datasets were generated or analyzed during the current study.

## References

[1] Mayrink J. and Reis Z. S. N. , Pre-Eclampsia in Low and Middle-Income Settings: What Are the Barriers to Improving Perinatal Outcomes and Evidence-Based Recommendations?, International Journal of Gynecology & Obstetrics. (2024) 164, no. 1, 33–39, 10.1002/ijgo.14913, 37329226. 37329226

[2] Akre S. , Sharma K. , Chakole S. , and Wanjari M. B. , Eclampsia and Its Treatment Modalities: A Review Article, Cureus. (2022) 14, no. 9, e29080, 10.7759/cureus.29080, 36249647. 36249647 PMC9555679

[3] Bögli S. Y. , Beqiri E. , Olakorede I. , Cherchi M. S. , Smith C. A. , Chen X. , Di Tommaso G. , Rochat T. , Tanaka Gutiez M. , Cucciolini G. , and Motroni V. , Unlocking the Potential of High-Resolution Multimodality Neuromonitoring for Traumatic Brain Injury Management: Lessons and Insights From Cases, Events, and Patterns, Critical Care. (2025) 29, no. 1, 10.1186/s13054-025-05360-4. PMC11956216 40165332

[4] Kazimierska A. , Uryga A. , Mataczyński C. , Burzyńska M. , Ziółkowski A. , Rusiecki A. , and Kasprowicz M. , Analysis of the Shape of Intracranial Pressure Pulse Waveform in Traumatic Brain Injury Patients, Annual International Conference of the IEEE Engineering in Medicine & Biology Society. (2021) 2021, 546–549, 10.1109/EMBC46164.2021.9630516, 34891352. 34891352

[5] Cucciolini G. , Motroni V. , and Czosnyka M. , Intracranial Pressure for Clinicians: It Is Not Just a Number, Journal of Anesthesia, Analgesia and Critical Care. (2023) 3, no. 1, 10.1186/s44158-023-00115-5. PMC10481563 37670387

[6] Robba C. , Pozzebon S. , Moro B. , Vincent J. L. , Creteur J. , and Taccone F. S. , Multimodal Non-Invasive Assessment of Intracranial Hypertension: An Observational Study, Critical Care. (2020) 24, no. 1, 10.1186/s13054-020-03105-z, 32591024. PMC7318399 32591024

[7] Csiba L. , Manual of Neurosonology, 2016, 1st edition, Cambridge University Press, 10.1017/CBO9781107447905.

[8] Berhanu D. , Ferreira J. C. , Abegão Pinto L. , Aguiar de Sousa D. , Lucas Neto L. , and Tavares F. J. , The Role of Optic Nerve Sheath Ultrasonography in Increased Intracranial Pressure: A Systematic Review and Meta Analysis, Journal of the Neurological Sciences. (2023) 454, no. 454, 120853, 10.1016/j.jns.2023.120853, 37925899. 37925899

[9] Rasulo F. A. , Calza S. , Robba C. , Taccone F. S. , Biasucci D. G. , Badenes R. , Piva S. , Savo D. , Citerio G. , Dibu J. R. , Curto F. , Merciadri M. , Gritti P. , Fassini P. , Park S. , Lamperti M. , Bouzat P. , Malacarne P. , Chieregato A. , Bertuetti R. , Aspide R. , Cantoni A. , McCredie V. , Guadrini L. , and Latronico N. , Transcranial Doppler as a Screening Test to Exclude Intracranial Hypertension in Brain-Injured Patients: The IMPRESSIT-2 Prospective Multicenter International Study, Critical Care. (2022) 26, no. 1, 10.1186/s13054-022-03978-2, 35428353. PMC9012252 35428353

[10] Robba C. , Picetti E. , Vásquez-García S. , Abulhasan Y. B. , Ain A. , Adeleye A. O. , Aries M. , Brasil S. , Badenes R. , Bertuccio A. , Bouzat P. , Bustamante L. , Calabro’ L. , Njimi H. , Cardim D. , Citerio G. , Czosnyka M. , Geeraerts T. , Godoy D. A. , Hirzallah M. I. , Devi B. I. , Jibaja M. , Lochner P. , Mijangos Méndez J. C. , Meyfroidt G. , Munusamy T. , Portilla J. P. , Prabhakar H. , Rasulo F. , Sánchez Parra D. M. , Sarwal A. , Shrestha G. S. , Shukla D. P. , Sung G. , Tirsit A. , Vásquez F. , Videtta W. , Wang Y. L. , Paiva W. S. , Taccone F. S. , and Rubiano A. M. , The Brussels Consensus for Non-Invasive ICP Monitoring When Invasive Systems Are Not Available in the Care of TBI Patients (the B-ICONIC Consensus, Recommendations, and Management Algorithm), Intensive Care Medicine. (2025) 51, no. 1, 4–20, 10.1007/s00134-024-07756-2, 39847066. 39847066

[11] Mastantuono J. M. , Combescure C. , Elia N. , Tramèr M. R. , and Lysakowski C. , Transcranial Doppler in the Diagnosis of Cerebral Vasospasm: An Updated Meta-Analysis, Critical Care Medicine. (2018) 46, no. 10, 1665–1672, 10.1097/CCM.0000000000003297, 30080684. 30080684

[12] Matta B. , Cucciolini G. , and Czosnyka M. , Cottrell J. E. , Patel P. M. , and Soriano S. G. , 7- Transcranial Doppler Ultrasonography in Anesthesia and Neurosurgery, Cottrell & Patel′s Neuroanesthesia, 2025, Seventh edition, Elsevier, 137–155, 10.1016/B978-0-323-93273-8.00007-9.

[13] Hirzallah M. I. , Lochner P. , Hafeez M. U. , Lee A. G. , Krogias C. , Dongarwar D. , Hartman N. D. , Ertl M. , Robba C. , Malojcic B. , Valaikiene J. , Sarwal A. , Hakimi R. , Schlachetzki F. , and for the Optic Nerve Sheath Diameter Point-of-Care Ultrasonography Quality Criteria Checklist (ONSD POCUS QCC) Expert Panelists , Optic Nerve Sheath Diameter Point-of-Care Ultrasonography Quality Criteria Checklist: An International Consensus Statement on Optic Nerve Sheath Diameter Imaging and Measurement∗, Critical Care Medicine. (2024) 52, no. 10, 1543–1556, 10.1097/CCM.0000000000006345, 38836697. 38836697

[14] van der Harst J. J. , Hijlkema J. , van der Werf S. , Elting J. W. J. , van Dijk J. M. C. , and Uyttenboogaart M. , Noninvasive Neurophysiological Diagnostics of Delayed Cerebral Ischemia in Aneurysmal Subarachnoid Hemorrhage: A Scoping Review, Acta Neurochirurgica, 2026, Springer, 10.1007/s00701-026-06901-8, 42115511. PMC13333557 42115511

[15] Rasulo F. A. , Togni T. , and Romagnoli S. , Essential Noninvasive Multimodality Neuromonitoring for the Critically Ill Patient, Critical Care. (2020) 24, no. 1, 10.1186/s13054-020-2781-2, 32204723. PMC7092614 32204723

[16] Godoy D. A. , Pérez-Bárcena J. , de Paula Delgado-Moya F. , Barea-Mendoza J. A. , and Llompart-Pou J. A. , Noninvasive Bedside Neuromonitoring in Acute Brain injury. A Narrative Review, Medicina Intensiva. (2025) 49, no. 12, 502305, 10.1016/j.medin.2025.502305. 40987633

[17] Patel S. , Maria-Rios J. , Parikh A. , and Okorie O. N. , Diagnosis and Management of Elevated Intracranial Pressure in the Emergency Department, International Journal of Emergency Medicine. (2023) 16, no. 1, 10.1186/s12245-023-00540-x. PMC10571389 37833652

[18] Carney N. , Totten A. M. , O′Reilly C. , Ullman J. S. , Hawryluk G. W. J. , Bell M. J. , Bratton S. L. , Chesnut R. , Harris O. A. , Kissoon N. , Rubiano A. M. , Shutter L. , Tasker R. C. , Vavilala M. S. , Wilberger J. , Wright D. W. , and Ghajar J. , Guidelines for the Management of Severe Traumatic Brain Injury, Fourth Edition, Neurosurgery. (2017) 80, no. 1, 6–15, 10.1227/NEU.0000000000001432, 27654000. 27654000

[19] Cavill G. , Simpson E. J. , and Mahajan R. P. , Factors Affecting Assessment of Cerebral Autoregulation Using the Transient Hyperaemic Response Test, British Journal of Anaesthesia. (1998) 81, no. 3, 317–321, 10.1093/bja/81.3.317, 9861111. 9861111

[20] Strunk D. , Veltkamp R. , Meuth S. G. , Chapot R. , and Kraemer M. , Intra-Arterial Application of Nimodipine in Reversible Cerebral Vasoconstriction Syndrome: A Neuroradiological Method to Help Differentiate From Primary Central Nervous System Vasculitis, Neurological Research and Practice. (2022) 4, no. 1, 10.1186/s42466-022-00173-0, 35227319. PMC8883624 35227319

[21] Nesheiwat O. and Al-Khoury L. , Reversible Cerebral Vasoconstriction Syndromes, 2025, StatPearls Publishing, http://www.ncbi.nlm.nih.gov/books/NBK551723/, 31869187. 31869187

[22] Ducros A. , Reversible Cerebral Vasoconstriction Syndrome, Lancet Neurology. (2012) 11, no. 10, 906–917, 10.1016/S1474-4422(12)70135-7, 22995694. 22995694

[23] Magid-Bernstein J. , Omran S. S. , Parikh N. S. , Merkler A. E. , Navi B. , and Kamel H. , Reversible Cerebral Vasoconstriction Syndrome: Symptoms, Incidence, and Resource Utilization in a Population-Based US Cohort, Neurology. (2021) 97, no. 3, e248–e253, 10.1212/WNL.0000000000012223, 34050007. 34050007 PMC8302148

[24] Rocha E. A. , Topcuoglu M. A. , Silva G. S. , and Singhal A. B. , RCVS(2) Score and Diagnostic Approach for Reversible Cerebral Vasoconstriction Syndrome, Neurology.(2019) 92, no. 7, e639–e647, 10.1212/WNL.0000000000006917, 30635475. 30635475

[25] Onichimowski D. , Nosek K. , Goraj R. , Jalali R. , Wińska A. , Pawlos A. , and Tuyakov B. , Use of Levosimendan in the Treatment of Cerebral Vascular Vasospasm: A Case Study, Drug Design, Development and Therapy. (2018) 12, 1777–1783, 10.2147/DDDT.S158237, 29950812. 29950812 PMC6018894

[26] Mathews N. G. , Cuschieri A. , Darwazeh M. , Sisi A. A. , Krishnan K. , and Darwazeh R. , Postpartum Intracerebral and Intraventricular Hemorrhage – A Case Report and Review of the Literature, Surgery Case Reports. (2025) 21, no. 6, 100142, 10.1016/j.sycrs.2025.100142.

[27] Kvisselgaard A. D. , Wolthers S. A. , Wikkelsø A. , Holst L. B. , Drivenes B. , and Afshari A. , Rapid Update and Revision of: Thromboelastography or Rotational Thromboelastometry Guided Algorithms in Bleeding Patients—An Updated Systematic Review With Meta-Analysis and Trial Sequential Analysis, Acta Anaesthesiologica Scandinavica. (2025) 69, no. 10, e70127, 10.1111/aas.70127, 41004381. 41004381

[28] Tang Y. , Yin F. , Fu D. , Gao X. , Lv Z. , and Li X. , Efficacy and Safety of Minimal Invasive Surgery Treatment in Hypertensive Intracerebral Hemorrhage: A Systematic Review and Meta-Analysis, BMC Neurology. (2018) 18, no. 1, 10.1186/s12883-018-1138-9, 30176811. PMC6120062 30176811

[29] Brasil S. , Patriota G. C. , Godoy D. A. , Paranhos J. L. , Rubiano A. M. , and Paiva W. S. , Monro-Kellie 4.0: Moving From Intracranial Pressure to Intracranial Dynamics, Critical Care. (2025) 29, no. 1, 10.1186/s13054-025-05476-7, 40474297. PMC12142851 40474297

[30] Bögli S. Y. , Olakorede I. , Beqiri E. , Cucciolini G. , Motroni V. , Smith C. A. , Cherchi M. S. , O′Leary R. , and Smielewski P. , Untangling Discrepancies Between Cerebrovascular Autoregulation Correlation Coefficients: An Exploration of Filters, Coherence and Power, Physiological Reports. (2025) 13, no. 8, e70332, 10.14814/phy2.70332, 40243158. 40243158 PMC12004267

[31] Beqiri E. , Ercole A. , Aries M. J. H. , Placek M. M. , Tas J. , Czosnyka M. , Stocchetti N. , Smielewski P. , CENTER-TBI High Resolution (HR ICU) Sub-Study Participants and Investigators , Anke A. , Beer R. , Bellander B. M. , Beqiri E. , Buki A. , Cabeleira M. , Carbonara M. , Chieregato A. , Citerio G. , Clusmann H. , Czeiter E. , Czosnyka M. , Depreitere B. , Ercole A. , Frisvold S. , Helbok R. , Jankowski S. , Kondziella D. , Koskinen L. O. , Kowark A. , Menon D. K. , Meyfroidt G. , Moeller K. , Nelson D. , Piippo-Karjalainen A. , Radoi A. , Ragauskas A. , Raj R. , Rhodes J. , Rocka S. , Rossaint R. , Sahuquillo J. , Sakowitz O. , Smielewski P. , Stocchetti N. , Sundström N. , Takala R. , Tamosuitis T. , Tenovuo O. , Unterberg A. , Vajkoczy P. , Vargiolu A. , Vilcinis R. , Wolf S. , Younsi A. , and Zeiler F. A. , Towards Autoregulation-Oriented Management After Traumatic Brain Injury: Increasing the Reliability and Stability of the CPPopt Algorithm, Journal of Clinical Monitoring and Computing. (2023) 37, no. 4, 963–976, 10.1007/s10877-023-01009-1, 37119323. 37119323 PMC10371880

[32] Vy V. and Goyal M. K. , Neurological Emergencies in Pregnant and Post-Partum Women in Resource-Poor Settings, Lancet Neurology. (2013) 12, no. 4, 329–330, 10.1016/S1474-4422(13)70021-8, 23518324. 23518324

[33] Collins L. , Lam L. , Kleinig O. , Proudman W. , Zhang R. , Bagster M. , Kovoor J. , Gupta A. , Goh R. , Bacchi S. , Schultz D. , and Kleinig T. , Verapamil in the Treatment of Reversible Cerebral Vasoconstriction Syndrome: A Systematic Review, Journal of Clinical Neuroscience. (2023) 113, 130–141, 10.1016/j.jocn.2023.05.013, 37267876. 37267876

[34] Papp Z. , Édes I. , Fruhwald S. , De Hert S. G. , Salmenperä M. , Leppikangas H. , Mebazaa A. , Landoni G. , Grossini E. , Caimmi P. , and Morelli A. , Levosimendan: Molecular Mechanisms and Clinical Implications: Consensus of Experts on the Mechanisms of Action of Levosimendan, International Journal of Cardiology. (2012) 159, no. 2, 82–87, 10.1016/j.ijcard.2011.07.022, 21784540. 21784540

[35] Hosford P. S. , Christie I. N. , Niranjan A. , Aziz Q. , Anderson N. , Ang R. , Lythgoe M. F. , Wells J. A. , Tinker A. , and Gourine A. V. , A Critical Role for the ATP-Sensitive Potassium Channel Subunit KIR6.1 in the Control of Cerebral Blood Flow, Journal of Cerebral Blood Flow & Metabolism. (2019) 39, no. 10, 2089–2095, 10.1177/0271678X18780602, 29862863. 29862863 PMC6775590

[36] Daoud H. A. S. , Kokoti L. , and Al-Karagholi M. A. M. , KATP Channels in Cerebral Hemodynamics: A Systematic Review of Preclinical and Clinical Studies, Frontiers in Neurology. (2024) 15, 1417421, 10.3389/fneur.2024.1417421, 39022739. 39022739 PMC11252034

[37] Kivikko M. , Kuoppamäki M. , Soinne L. , Sundberg S. , Pohjanjousi P. , Ellmen J. , and Roine R. O. , Oral Levosimendan Increases Cerebral Blood Flow Velocities in Patients With a History of Stroke or Transient Ischemic Attack: A Pilot Safety Study, Current Therapeutic Research. (2015) 77, 46–51, 10.1016/J.CURTHERES.2015.01.001, 26082815. 26082815 PMC4461857

[38] Riley D. S. , Barber M. S. , Kienle G. S. , Aronson J. K. , von Schoen-Angerer T. , Tugwell P. , Kiene H. , Helfand M. , Altman D. G. , Sox H. , Werthmann P. G. , Moher D. , Rison R. A. , Shamseer L. , Koch C. A. , Sun G. H. , Hanaway P. , Sudak N. L. , Kaszkin-Bettag M. , Carpenter J. E. , and Gagnier J. J. , CARE Guidelines for Case Reports: Explanation and Elaboration Document, Journal of Clinical Epidemiology. (2017) 89, 218–235, 10.1016/j.jclinepi.2017.04.026, 28529185. 28529185

