# Supplementary material for: Multimodal Monitoring in Eclamptic Intracerebral Hemorrhage Complicated by Reversible Cerebral Vasoconstriction Syndrome (RCVS): A Case Report
Source: Case Rep Crit Care. 2026 Aug 27;2026:7731993. doi: 10.1155/crcc/7731993 (PMC13519953; doi:10.1155/crcc/7731993)
Supplement: Supplementary file 1 [file CRCC-2026-7731993-s001.pdf]

CARE Checklist of information to include when writing a case report

CARE case report guidelines

(cc) BY-NC-ND

Topic	Item	Checklist item description	Reported on Line
Title	1	The diagnosis or intervention of primary focus followed by the words "case report"	1-5
Key Words	2	2 to 5 key words that identify diagnoses or interventions in this case report, including "case report"	45
Abstract (no references)	3a	Introduction: What is unique about this case and what does it add to the scientific literature?	36-37
	3b	Main symptoms and/or important clinical findings	39
	3c	The main diagnoses, therapeutic interventions, and outcomes	39,42, 47,50, 54-55
	3d	Conclusion—What is the main "take-away" lesson(s) from this case?	56-59
Introduction	4	One or two paragraphs summarizing why this case is unique (**may include references**)	84-88
Patient Information	5a	De-identified patient specific information.	90
	5b	Primary concerns and symptoms of the patient.	91
	5c	Medical, family, and psycho-social history including relevant genetic information	92-93
	5d	Relevant past interventions with outcomes	92-94
Clinical Findings	6	Describe significant physical examination (PE) and important clinical findings.	91-97
Timeline	7	Historical and current information from this episode of care organized as a timeline	from 90
Diagnostic Assessment	8a	Diagnostic testing (such as PE, laboratory testing, imaging, surveys).	94,95,100,104,106,125,137,142
	8b	Diagnostic challenges (such as access to testing, financial, or cultural)	NA
	8c	Diagnosis (including other diagnoses considered)	95-97,108,126,139
	8d	Prognosis (such as staging in oncology) where applicable	NA
Therapeutic Intervention	9a	Types of therapeutic intervention (such as pharmacologic, surgical, preventive, self-care)	97,104,120,127,139-140
	9b	Administration of therapeutic intervention (such as dosage, strength, duration)	139-140
	9c	Changes in therapeutic intervention (with rationale)	NA
Follow-up and Outcomes	10a	Clinician and patient-assessed outcomes (if available)	145-152
	10b	Important follow-up diagnostic and other test results	145-152
	10c	Intervention adherence and tolerability (How was this assessed?)	NA
	10d	Adverse and unanticipated events	NA
Discussion	11a	A scientific discussion of the strengths AND limitations associated with this case report	
	11b	Discussion of the relevant medical literature **with references**.	155-192
	11c	The scientific rationale for any conclusions (including assessment of possible causes)	
	11d	The primary "take-away" lessons of this case report (without references) in a one paragraph conclusion	194-197
Patient Perspective	12	The patient should share their perspective in one to two paragraphs on the treatment(s) they received	NA
Informed Consent	13	Did the patient give informed consent? Please provide if requested	Yes ☑ No ☐
